# *Enterobacter* sp. AA26 as a Protein Source in the Larval Diet of *Drosophila suzukii*

**DOI:** 10.3390/insects12100923

**Published:** 2021-10-09

**Authors:** Katerina Nikolouli, Fabiana Sassù, Spyridon Ntougias, Christian Stauffer, Carlos Cáceres, Kostas Bourtzis

**Affiliations:** 1 Insect Pest Control Laboratory, Joint FAO/IAEA Centre of Nuclear Techniques in Food and Agriculture, Department of Nuclear Sciences and Applications, IAEA Laboratories, 2444 Seibersdorf, Austria; fabiana.sassu@gmail.com (F.S.); c.e.caceres-barrios@iaea.org (C.C.); k.bourtzis@iaea.org (K.B.); 2 Department of Forest and Soil Sciences, Boku, University of Natural Resources and Life Sciences, 1190 Vienna, Austria; christian.stauffer@boku.ac.at; 3 Roklinka 224, Dolní Jirčany, 252 44 Psáry, Czech Republic; 4 Laboratory of Wastewater Management and Treatment Technologies, Department of Environmental Engineering, Democritus University of Thrace, Vas. Sofias 12, 67100 Xanthi, Greece; sntougia@env.duth.gr

**Keywords:** spotted-wing drosophila, symbiotic bacteria, gut microbiota, pest-management, mass-rearing, insect fitness

## Abstract

**Simple Summary:**

*Drosophila suzukii* has caused considerable damages to a variety of soft fruit crops. The sterile insect technique (SIT) is one of the most promising candidates that has recently attracted significant research efforts. A SIT package requires highly productive and cost-efficient mass rearing of the insects to produce a sufficient amount of males that will be sterilized and consequently released in the target area. Operational costs of mass-rearing facilities can be remarkably high, mainly due to the larval diet used for rearing. Gut symbiotic bacteria have been shown to enhance the productivity and development of fruit flies when used as supplements or protein source of their larval diet. In this study, we evaluated whether *Enterobacter* sp. AA26 could replace inactive brewer’s yeast as a protein source in *D. suzukii* larval diet and effects on the biological quality of the flies are discussed.

**Abstract:**

The Spotted-Wing Drosophila fly, *Drosophila suzukii*, is an invasive pest species infesting major agricultural soft fruits. *Drosophila suzukii* management is currently based on insecticide applications that bear major concerns regarding their efficiency, safety and environmental sustainability. The sterile insect technique (SIT) is an efficient and friendly to the environment pest control method that has been suggested for the *D. suzukii* population control. Successful SIT applications require mass-rearing of the strain to produce competitive and of high biological quality males that will be sterilized and consequently released in the wild. Recent studies have suggested that insect gut symbionts can be used as a protein source for *Ceratitis capitata* larval diet and replace the expensive brewer’s yeast. In this study, we exploited *Enterobacter* sp. AA26 as partial and full replacement of inactive brewer’s yeast in the *D. suzukii* larval diet and assessed several fitness parameters. *Enterobacter* sp. AA26 dry biomass proved to be an inadequate nutritional source in the absence of brewer’s yeast and resulted in significant decrease in pupal weight, survival under food and water starvation, fecundity, and adult recovery.

## 1. Introduction

The gut of insects is the receptacle of a rich diversity of symbiotic bacteria, which can influence nutrient assimilation, host physiology, biology and ecology, including sexual and social behavior, and fitness [1,2,3,4,5]. The study of these microorganisms has brought to light mechanisms of symbiotic interactions that have proved to be a source of knowledge to manipulate insect behavior and develop microbe-based pest control techniques i.e., repellents, attract-and-kill, and mass trapping [6,7]. Recently gut microbiota have been exploited to increase the efficacy of the sterile insect technique (SIT) [8,9]. The SIT is an environment-friendly approach for population management with appreciable economic and social benefits that has been effectively applied worldwide on different insect pests [10,11]. SIT highly relies on efficient mass-rearing methods allowing the continuous production of large numbers of high-quality insects [12]. Insects (preferentially males) are then sterilized by irradiation and released with an overflow ratio into a target area, where they are expected to compete with wild males and mate with wild females [12]. Successful matings between sterile males and wild females will not produce viable offspring that eventually cause the intended population to decline in the next generations [10]. 

One of the most significant parts of a SIT programme is the larval diet used during insect mass-rearing. The artificial diet is one of the most critical and expensive components for the production of high-quality sterile insects and its development is guided by the availability of ingredients, nutritional value, ease of storage, labour and cost [13,14]. The diet used for mass-reared insects should supply proper and sufficient nutritional factors allowing for an efficient maturation into the adult stage and high adult fitness [13,15]. In addition, the components of the mass-rearing diet should be cost-effective without any side effects on insect fitness and overall colony productivity [13,16]. In the long run, diet expenses and insect performance should be well-balanced to achieve highly competitive insects at a reduced cost.

Several studies have focused on exploiting insect gut symbiotic bacteria as probiotics or alternative protein sources to enhance the SIT of fruit flies [17,18,19,20,21,22,23,24,25,26,27,28]. In the case of *Ceratitis capitata* (Wiedemann) (Diptera: Tephritidae), enrichment of the larval diet with live bacteria of the *Enterobacteriacae* family shortened the immature development stages, extended survival and improved the male mating competitiveness [9,17,21,22,23,24]. Increased fecundity was also observed when *Bactrocera oleae* (Rossi) (Diptera: Tephritidae) was fed with *Enterobacteriacae* microbiota [27,29]. Similarly, bacteria such as *Candidatus* Erwinia dacicola [30] and *Pseudomonas putida*, which are common in the wild population of *Bactrocera oleae* (Rossi) (Diptera: Tephritidae), were found beneficial for larval development and female fecundity, respectively [27,29].

In all the above studies the beneficial probiotic effect was shown by inoculating the diet with live bacteria while the use of inactive bacteria is limited [18,28]. Kyritsis and colleagues [28] assessed whether the use of inactive *Enterobacter* sp. AA26 biomass could be used in the larval diet of *C. capitata* as an alternative protein source to replace the costly brewer’s yeast. Incorporating dead *Enterobacter* sp. AA26 benefited substantially the biological quality and productivity of reared *C. capitata* and paved the way to explore the use of inactive bacteria as the main protein source in the larval diet.

*Drosophila suzukii* (Diptera: Drosophilidae) is one of the most damaging insect pests of soft skinned fruits in North America and Europe in the last decade, but also a newly introduced detrimental insect in South America and Africa [31,32,33,34]. The also-called spotted wing Drosophila (SWD) fly can damage a wide range of economically important soft-skin fruits that are easily perforated by the females sclerotized ovipositor to lay their eggs [35,36]. Eggs hatch and larvae feed on the fruit pulp which causes the total rot of the fruit and an economic impact that accounts for millions of revenue losses each year [37]. *Drosophila suzukii* infestations are primarily controlled by the application of various insecticides, but the rising concerns on their poor effectiveness and possible impacts on the health of farmers and consumers have initiated the development of eco-friendly pest management tactics such as netting and tunneling, attract-and-kill baits deployment, alternative oviposition sites, and parasitoid releases [38,39]. Researchers have recently begun to evaluate SIT as a strategy to control *D. suzukii* populations in confined areas [40,41,42]. Currently, species-specific protocols of irradiation, mass-rearing, packaging, and quality control are fully or partially available for the implementation of this technique to control *D. suzukii* populations [42,43,44]. Nevertheless, the use of probiotics in *D. suzukii*’s larval or adult diet can help to further improve some of these protocols i.e., increase the mass-rearing productivity and/or boost the quality of the released sterile flies. 

Previous studies have characterized bacterial species frequently occurring within the gut of *D. suzukii*, in some cases as an attempt to improve the effectiveness of bait traps used for its population management [45,46], and in others to understand its peculiar specialization in ripe and ripening fruits compared to other drosophilid species [47,48,49,50]. As a result, bacterial families such as *Enterobacteriaceae, Acetobacteraceae, Lactobacillaceae*, and *Enterococcaceae,* which are generally observed in association with different wild or laboratory-reared species of drosophilid [51], were also found in *D. suzukii* [52,53]. Several studies have identified *Enterobacteriaceae* to be abundant in *Drosophila melanogaster* [51,54,55] and *D. suzukii* [47,49,50,52,53,56]. Martinez- Sañudo et al. observed that *Enterobacteriaceae* in *D. suzukii* were more abundant and diverse in newly colonized areas compared to flies adapted in the new habitat [53]. Interestingly, *Enterobacter* sp. has been identified in few *D. suzukii* studies with varying diversity and abundance [52,53,57]. 

Distinct microbial populations have been correlated with positive impacts on fly longevity and development [49,58]. Bing et al. showed that *D. suzukii* utilizes microbes as a source of protein when reared on fruit-based diets [49]. During undernutrition *D. melanogaster* can use a wide range of microbes which serve as a protein source [58]. However, it is not yet clear how all fly-associated microbes contribute to nutrition. 

A comparative study between *Enterobacter* sp. AA26 and Torula yeast (a yeast type that is different than brewer’s yeast) showed that *Enterobacter* sp. AA26 was equivalent to yeast in terms of nutritional value [59]. The biomass of the strain AA26 provided all the essential and non-essential amino acids and vitamins required for the development of *C. capitata* larvae. Considering the abundance of *Enterobacteriaceae* species in fruit flies, including *D. suzukii*, the beneficial effects of *Enterobacter* sp. AA26 on the mass-reared *C. capitata*, the high protein content of *Enterobacter* sp. AA26 and the role of microbes as protein sources for *Drosophila* flies, we evaluated *Enterobacter* sp. AA26 as a potential protein source that could totally or partially replace inactive brewer’s yeast in the *D. suzukii* larval diet. Assessing the potential beneficial effect of the same bacteria species in different insect species is of critical importance because if results are positive, they will justify economically the insect production in mass-rearing facilities. We assessed the effect of *Enterobacter* sp. AA26 on several fitness parameters, including pupal weight and recovery, adult emergence rate, sex ratio, flight ability, adult survival under stress and female fecundity. These fitness parameters are considered production and quality indicators of flies used for SIT programmes and ensure that insects of poor quality that can lead to a lack of effective control and higher programme costs are excluded [60].

## 2. Materials and Methods

### 2.1. Drosophila Suzukii Rearing Colony 

All experiments were performed using the *D. suzukii* colony maintained at the Insect Pest Control Laboratory (IPCL) of the Joint FAO/IAEA Centre of Nuclear Techniques in Food and Agriculture, Seibersdorf, Austria. The colony was obtained from the Agricultural Entomology Unit of the Edmund Mach Foundation in San Michele All’Adige, Trento, Italy. Adult flies were maintained in metal-framed and mesh-covered cages of 45 × 45 × 45 cm under controlled laboratory temperature, RH and light conditions (22 ± 5 °C, 65 ± 5% RH, and 14:10 h light:dark (L:D) photoperiod). 

### 2.2. Enterobacter *sp. AA26* Biomass Production and Larval Diet Preparation

Inactive *Enterobacter* sp. AA26 biomass was produced as described in Kyritsis et al. [28]. Briefly, a 1 L laboratory-scale bioreactor was used to grow *Enterobacter* sp. AA26 under aseptic conditions. The bioreactor was fed with Luria-Bertani (LB) broth and operated under the fill and draw mode. Adequate aeration of the bioreactor was achieved through an air pump and continuous agitation. The bacterial biomass was collected by centrifugation at 4000× *g* for 10 min and stored at −80 °C until use.

The biomass was dried at 60 °C for 24 h and subsequently grinded in a Planetary Ball Mill PM100 for 3.5 min, until a fine powder was obtained. The rearing larval diet, consisting of 28% wheat bran, 7% inactive brewer’s yeast (*Saccharomyces cerevisiae*), 13% sugar, 0.45% sodium benzoate, 0.45% nipagin, and 51% water, was used as a standard control diet. All solid components (apart from nipagin) were weighted and mixed into water to a total volume of 1 L. The solution was constantly stirred and brought to boil. It was left simmering for 10 min and then cooled slightly for an additional 10 min. Nipagin was diluted in 10 mL water, added to the media and mixed well. To evaluate the effect of yeast replacement with *Enterobacter* sp. AA26, we used 1) a full replacement diet (hereafter as “total”) containing 7% *Enterobacter* sp. AA26 biomass instead of inactive brewer’s yeast, and a partial replacement diet with 3.5% inactive brewer’s yeast and 3.5% *Enterobacter* sp. AA26 (hereafter as “partial”). We decided to use the 1:1 *Enterobacter* sp. AA26:yeast ratio based on the similar study performed in medfly [28]. 

### 2.3. Drosophila Suzukii Egg Collection

Eggs used for the experiments were obtained following the wax-rearing procedure developed at the IPCL [44]. Eggs were collected from the mass-rearing colony of the IPCL and placed on moist filter paper to avoid desiccation. All eggs were collected within a period of 4 h to prevent sample variation. Twenty-four hours after the collection, eggs were transferred on a wet black net in a Petri dish (size: 70 × 15 mm (D × H)) containing about 150 g of diet. Four replicates of 500 eggs each were collected per treatment (i.e., *n* = 2000 eggs per treatment). These eggs were used to evaluate pupal weight and recovery, adult emergence rate, sex ratio, flight ability, adult survival under stress and female fecundity. The number of replicates used in each experiment are shown below in the respective M&M section.

### 2.4. Effect of Enterobacter *sp. AA26* on Pupae and Adult Recovery 

To assess the pupae and adult recovery, we measured number of pupae, adult emergence and sex ratio. Ten days after the egg collection, all pupae were removed from the diet and counted. All pupae that recovered from the same larval treatment (4 petri dishes per treatment) were placed on moist paper and left in boxes until adult emergence in a room with regulated temperature, humidity and light. Adult emergence was recorded daily using CO_2_ anesthesia. The sex ratio was determined as proportion of males per total number of adults. Adult emergence and sex ratio data were also collected from the flight ability and survival under stress experiments and all data coming from the different assays were combined for statistical analysis. 

### 2.5. Effect of Enterobacter *sp. AA26* on Pupal Weight 

To determine the pupal weight, dark-brown pupae 24 h before emergence were selected from each treatment. Until the day of the experiment pupae were maintained in a room with regulated temperature, humidity and light to avoid any bias in the weight data. Pupal weight was determined by weighing independently pools of 10 pupae. Each single pool represented one sample. For each treatment (partial, total, and control), we performed 12 replicates (*n* = 120 pupae per treatment). All data were combined for statistical comparisons. 

### 2.6. Effect of Enterobacter *sp. AA26* on Survival

The adult survival was tested under food and water deprivation. Two days before emergence, 100 pupae from each treatment were randomly collected and individually placed into a 96-well microtiter plate sealed with plastic film. Pupae were checked twice a day to record the time of emergence, the time of death, and the sex of each fly. The film on the top of each well was delicately perforated to allow air exchange. Plates were kept in the dark to reduce the mobility at standard laboratory conditions. One plate was set up for each treatment. Each single adult represented one sample.

### 2.7. Effect of Enterobacter *sp. AA26* on Flight Ability

The flight ability test was performed following the standard Quality Control Procedures applied to evaluate the sterile insects used in SIT applications. Pupae from each treatment were placed within a ring of paper centered in the bottom of the Petri dish to allow newly emerged flies a place to rest. A black plexiglass tube was placed over the Petri dish and was lightly coated inside with unscented talcum powder to prevent the flies from walking out. Flies that emerged were periodically removed from the vicinity of the tubes to minimize fly-back or fall-back into the tubes. For each treatment four replicates were conducted each consisting of 30 pupae (*n* = 120 pupae per treatment).

### 2.8. Effect of Enterobacter *sp. AA26* on Female Fecundity

Newly emerged adults were selected from each treatment, sexed and separated into “triplets” made of one female and two males to ensure female insemination. Triplets were then transferred into 200 cm^3^ volume rectangular plexiglass cages and were provided with water and standard adult diet under the standard laboratory conditions. Twenty replicates were performed for each of the treatments and the control group. After 72 h, males were discarded, and females were individually placed into a Petri dish provided with raspberry-juice agar substrate as egg oviposition site. Oviposition was allowed for 48 h without any interruption, after which the females were discarded, and the number of laid eggs was counted. Each single female represented a replicate. Females that did not lay any eggs or laid fewer than 10 eggs were not included in the analysis.

### 2.9. Statistical Analyses

All statistical analyses were performed using R version 4.0.5 [61]. 

Pupal weight: Pupal weight data are continuous variables and therefore assume a normal distribution. A linear model was applied for their analysis.

Pupae and adults recovery: The number of pupae and adults recovered per treatment are count data and were analyzed with GLM with negative binomial distribution and a log link function. Negative binomial was applied due to overdispersion detected in the Poisson and the Quasi-Poisson GLM models [62,63]. Analysis of deviance was performed with an F-test [64]. 

Sex ratio (proportion males): Sex ratio proportional data assume a binomial distribution and were analyzed with a GLM-binomial family and a logit link function [65]. Analysis of deviance was performed with a Chi-squared test [64]. 

Adult survival: The survivorship curves were calculated using a Kaplan–Meier approach (survfit package) [66]. The package survival was used for modeling the survival data [67].

Flight ability: Rate of fliers are proportional data and assume a binomial distribution [65]. Data were analyzed with a GLM-binomial family and a logit link function. Analysis of deviance was performed with a Chi-squared test [64]. 

Fecundity: Fecundity data are count data and were analyzed with a generalized linear model (GLM) with negative binomial distribution and a log link function. Negative binomial was applied due to overdispersion detected in the Poisson model [62,63]. Analysis of deviance was performed with an F-test [64].

Residuals of the models were checked for normality and homogeneity of variance. Goodness-of-fit of the models was visually inspected with half-normal plots with simulation envelopes [68]. In addition, analysis of variance was performed for each model to check assess differences in the fit statistics [65]. Overdispersion of the generalized linear models was checked with the DHARMA package [69]. DHARMA tests if the simulated dispersion is equal to the observed dispersion and supports the visual inspection of the residuals. Lsmeans package were used for the pair-wise comparisons of the fitted model estimates [70]. In all cases, the mean ± standard error is reported. In all boxplots, both the mean and the median values are depicted. Significant differences between treatment groups are indicated in the boxplots with asterisks (*** *p* ≤ 0.001, ** *p* ≤ 0.01, * *p* ≤ 0.05, ns: *p* > 0.05; confidence level used: 0.95, alpha = 0.05). Non-significant differences are not shown in the boxplots.

## 3. Results

### 3.1. Effect of Enterobacter *sp. AA26* on Pupal Weight 

**Figure 1:**
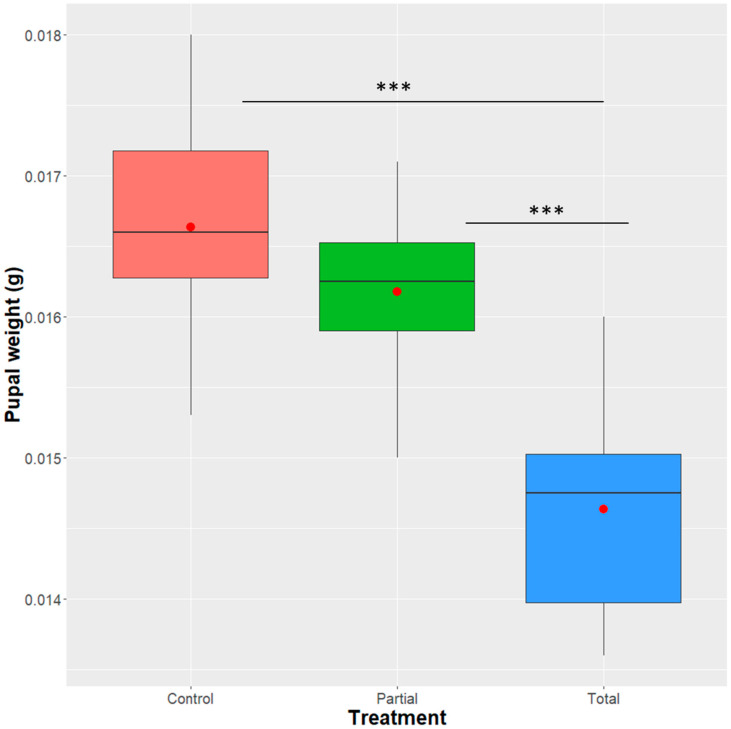
Effect of *Enterobacter* sp. AA26 on pupal weight. Data were analyzed with a linear model to define if the different treatments had a significant effect on the average pupal weight. Boxplots span the interquartile range and whiskers indicate the highest and lowest observations. The line and the dot inside each box represent the median and the mean, respectively. Significant differences between treatment groups are indicated with asterisks (*** *p* ≤ 0.001, ** *p* ≤ 0.01, * *p* ≤ 0.05, ns: *p* > 0.05; confidence level used: 0.95, alpha = 0.05).

Brewer’s yeast replacement with *Enterobacter* sp. AA26 had a significant effect on the average weight of the pupae (linear model: F = 26.45, df = 2, 33, *p* = 1.395 × 10^−7^) (average pupal weight: total: 0.0146 g ± 0.0007; partial: 0.0162 g ± 0.0006; control: 0.0166 g ± 0.0008). Pairwise comparisons among the tested diets (total, partial, control) indicated that the pupal weight was significantly lower in the total replacement when compared either with the partial or the control treatments (t-value = 5.351, *p* = < 0.0001; and t-value = 6.942, *p* = < 0.0001, respectively), while, between the partial and the control treatments, the difference was not significant (t-value = 1.591, *p* = 0.1212) (Figure 1).

### 3.2. Effect of Enterobacter *sp. AA26* on Developmental Parameters

#### 3.2.1. Pupae Recovery 

**Figure 2:**
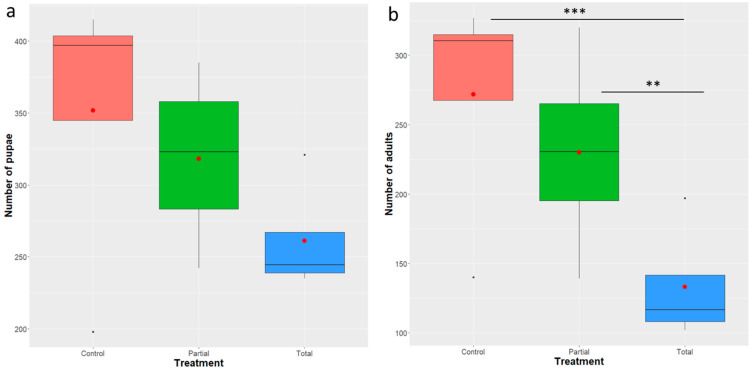
Effect of *Enterobacter* sp. AA26 on pupal and adult recovery. A GLM (negative binomial family) was used to analyze the impact of the various treatments on the recovery of pupae and adults; (**a**). Number of pupae recovered per treatment; (**b**). Number of adults recovered per treatment. Boxplots span the interquartile range and whiskers indicate the highest and lowest observations. The line and the dot inside each box represent the median and the mean, respectively. Significant differences between treatment groups are indicated with asterisks (*** *p* ≤ 0.001, ** *p* ≤ 0.01, * *p* ≤0.05, ns: *p* > 0.05; confidence level used: 0.95, alpha = 0.05).

The mean number of pupae recovered per treatment grown in the total replacement diet was 261 ± 20.15 compared to 318 ± 31.2 and 352 ± 51.5 of the partial and control treatments, respectively (Figure 2a). Analysis of deviance indicated that treatment is not a significant predictor for pupal recovery (GLM negative binomial model: F = 2.0914, df = 2, 9, *p =* 0.123) based on the GLM-negative binomial model. A marginal significant difference was detected at the pairwise comparison of the total and partial treatments (z = 2.026, *p* = 0.0427). 

#### 3.2.2. Adult Recovery and Sex Ratio

Analysis of deviance indicated that treatment is a significant predictor for the number of adults recovered (GLM negative binomial model: F = 5.9898, df = 2, 9, *p* = 0.002504). Total replacement of brewer’s yeast with *Enterobacter* sp. AA26 significantly decreased the adult recovery compared to both the partial replacement and the control treatment (z = 2.610, *p* = 0.0090 and z = 3.416, *p* = 0.0006, respectively) (Figure 2b). We did not observe any significant adult recovery decrease between the partial replacement and the control treatment (z = 0.808, *p* = 0.4190) (Figure 2b).

**Figure 3:**
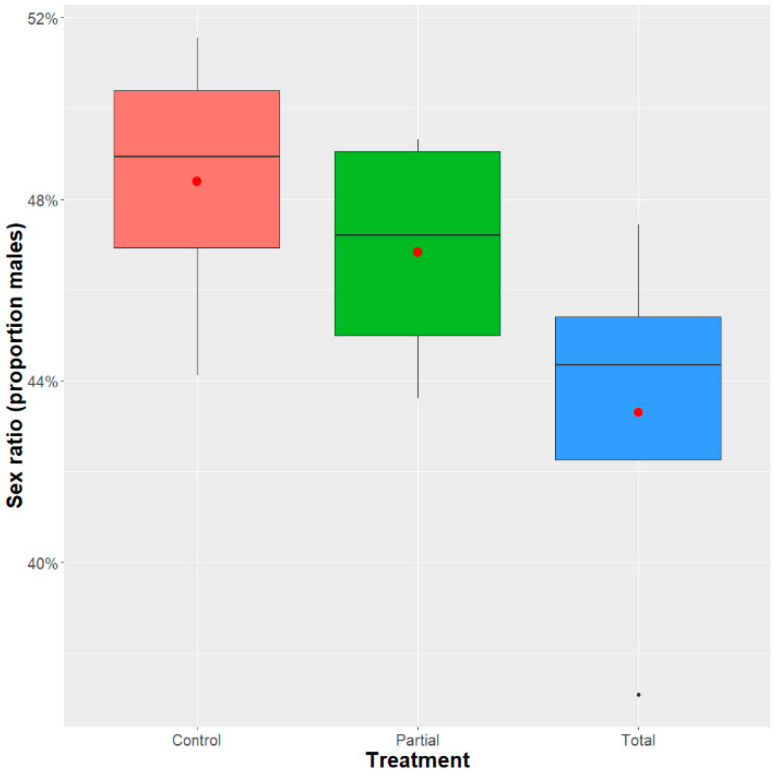
Sex ratio of males developed in the partial and total replacement of *Enterobacter* sp. AA26 diet and control diet. Sex ratio was determined as the percentage of males per total number of adults. A GLM (binomial family) analysis was performed to determine the effect of *Enterobacter* sp. AA26 diet replacement. Boxplots span the interquartile range and whiskers indicate the highest and lowest observations. The line and the dot inside each box represent the median and the mean, respectively. Significant differences between treatment groups are indicated with asterisks (*** *p* ≤0.001, ** *p* ≤ 0.01, * *p* ≤ 0.05, ns: *p* > 0.05; confidence level used: 0.95, alpha = 0.05).

The partial and total replacement of *Enterobacter* sp. AA26 did not affect the sex ratio of the emerged adults (GLM binomial model: analysis of deviance: χ^2^ = 4.8549, df = 9, *p* = 0.218) (Figure 3).

### 3.3. Effect of Enterobacter *sp. AA26* on Adult Survival under Food and Water Starvation

**Figure 4:**
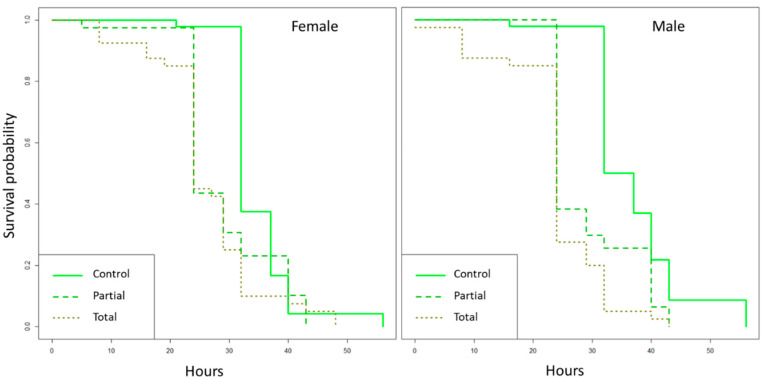
Effect of *Enterobacter* sp. AA26 on female (**left**) and male (**right**) survival under starvation. Significant differences were measured with a log-rank test. The x-axis represents time in hours.

The presence of *Enterobacter* sp. AA26 had a negative impact on the *D. suzukii* adult survival under food and water starvation (♀log-rank test: χ^2^ = 19, df = 2, *p* = 7 × 10^−5^; ♂ log-rank test: χ^2^ = 40.5, df = 2, *p* = 2 × 10^−9^). Males developed in the total replacement diet had significantly shorter survival times compared to the ones developed in the partial and the control diets (log-rank test: χ^2^ = 5.7, df = 1, *p* = 0.02; log-rank test: χ^2^ = 15.8, df = 1, *p* = 7 × 10^−5^, respectively). In the case of females, the survival probability was significantly shorter when grown in the total replacement diet compared to the control one (log-rank test: χ^2^ = 18.9, df = 1, *p* = 1 × 10^−5^), but no significant difference was detected between the partial and the control diets (log-rank test: χ^2^ = 0.4, df = 1, *p* = 0.5) (Figure 4).

### 3.4. Effect of Enterobacter *sp. AA26* on Flight Ability

**Figure 5:**
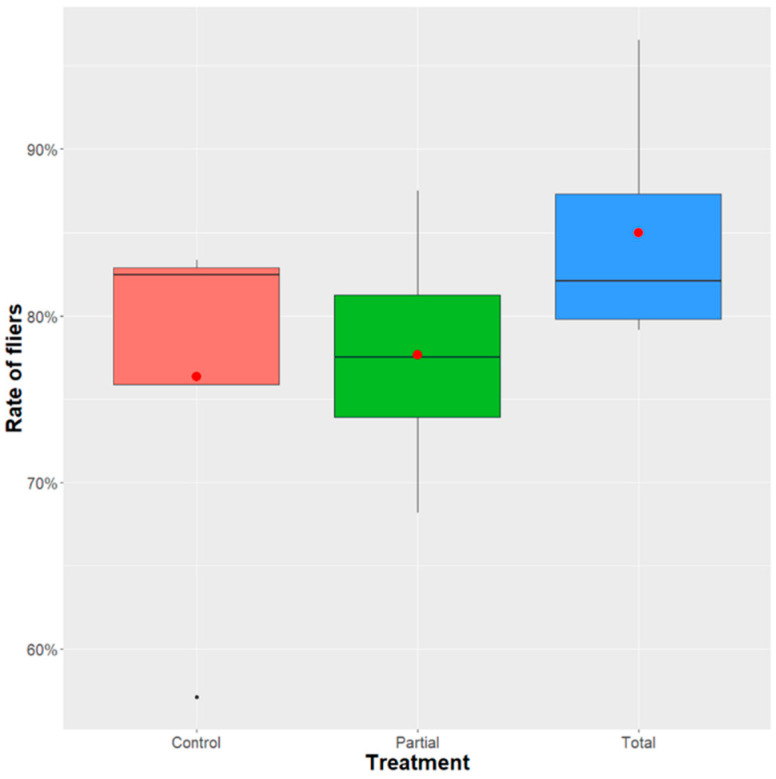
Flight ability of *D. suzukii* adults. The rate of fliers was calculated based on the emerged pupae. A GLM (binomial family) analysis was performed to determine the effect of *Enterobacter* sp. AA26 diet replacement. Boxplots span the interquartile range and whiskers indicate the highest and lowest observations. The line and the dot inside each box represent the median and the mean, respectively. Significant differences between treatment groups are indicated with asterisks (*** *p* ≤ 0.001, ** *p* ≤ 0.01, * *p* ≤0.05, ns: *p* > 0.05; confidence level used: 0.95, alpha = 0.05).

The addition of *Enterobacter* sp. AA26 in the *D. suzukii* larval diet did not affect the adult flight ability (GLM binomial model-analysis of deviance: χ^2^ = 14.829, df = 9, *p* = 0.1942). The rate of fliers was calculated based on the emerged pupae and data showed that there was no significant difference among the three treatments. The rate of fliers was 76.3% ± 1.28, 77.7% ± 0.80, and 85% ± 0.80 for the total, partial and control treatments, respectively (Figure 5).

### 3.5. Effect of Enterobacter *sp. AA26* on Fecundity

**Figure 6:**
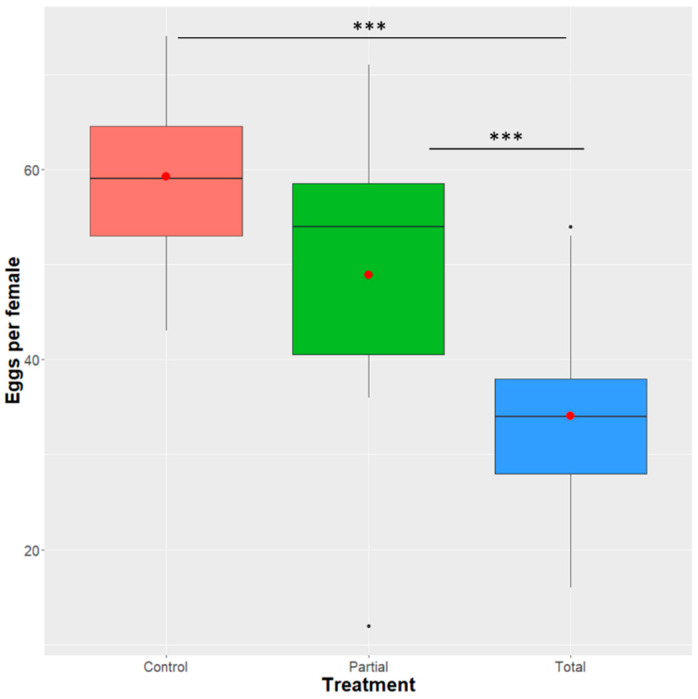
Effect of *Enterobacter* sp. AA26 on *D. suzukii* female fecundity. The number of eggs per female are shown in the y-axis for each of the treatment groups and control group. Analysis of fecundity were performed using a GLM (negative binomial family). Boxplots span the interquartile range and whiskers indicate the highest and lowest observations. The line and the dot inside each box represent the median and the mean, respectively. Significant differences between treatment groups are indicated with asterisks (*** *p* ≤ 0.001, ** *p* ≤ 0.01, * *p* ≤ 0.05, ns: *p* > 0.05; confidence level used: 0.95, alpha = 0.05).

Analysis of deviance indicated that treatment is a significant predictor for the egg production of *D. suzukii* females (GLM negative binomial model: F = 15.263, df = 2, 37, *p =* 2.352 × 10^−7^). The mean number of eggs per female grown in the total replacement diet (22.1 ± 3.88) was significantly lower compared to the partial (35.9 ± 6.76) and the control diets (44.7 ± 6.01) (total-partial: z = 3.299, *p* = 0.0010; total-control: z = 5.516, *p* < 0.0001). The model did not detect a significant difference between the partial and the control treatments (z = 1.848, *p* = 0.0646) (Figure 6). 

## 4. Discussion

In our study, results indicate that *Enterobacter* sp. AA26 dry biomass is not adequate to fully replace inactive brewer’s yeast as a protein source in *D. suzukii* larval diet. In particular, complete replacement of brewer’s yeast resulted in significant decrease in pupal weight, survival under food and water starvation, fecundity, and adult recovery. In addition, neither the partial nor the complete replacement of yeast with *Enterobacter* sp. AA26 had any significant impact on flight ability, sex ratio, and pupal recovery. 

Core aspects of insect physiology are influenced or regulated by gut microbial communities by promoting digestive activities, boosting immune responses and restricting pathogen colonization [71]. Several studies have characterized the diverse microbial communities harboring the gut of natural *Drosophila* populations, with *Enterobacteriaceae* being one of the predominant families [51,72,73]. Studies on *D. suzukii* also confirmed the presence of *Enterobacteriaceae* in their digestive tract [52,53]. *Enterobacter* belongs to the *Enterobacteriaceae* family and is considered one of the most dominant genera of the gut of several insect species [74,75,76,77]. Due to their pivotal role in host physiology and biology, *Enterobacter* spp. could be exploited for the control of pest species. Recent studies have explored the possibility of using *Enterobacter* spp. as probiotic supplement in larval diets of mass-rearing insects for large scale operational SIT programs [22,23,24,28]. *Enterobacter* sp. AA26 was isolated from the gut of *C. capitata* males and females and assessed for its probiotic effects on several fitness parameters [23,24]. Results in *C. capitata* indicated that addition of *Enterobacter* sp. AA26 increased pupae and adult production and decreased rearing duration for several developmental stages. On the other hand, pupal weight, sex ratio, male mating competitiveness, flight ability or life span under food and water deprivation were not affected. *Enterobacter* sp. AA26 dry biomass was also tested as a potential protein source that could fully replace the brewer’s yeast in the *C. capitata* larval diet [28]. *Enterobacter* sp. AA26 proved to be an adequate nutritional source for *C. capitata* larvae since the immature stages’ mortality decreased, the pupae development was accelerated, the pupal weight increased and the adult survival under food and water deprivation was elongated. 

*Drosophila suzukii* is a continuously expanding threat and a SIT-based approach has been proposed as a promising control option. Mass-rearing protocols and the larval diet are important aspects that will determine the production of insects of high biological quality that will be released in the target area. *Drosophila suzukii* diet is currently based on brewer’s yeast as a protein source. Previous studies have pointed out the critical role of yeast for *D. suzukii* as a source of dietary protein that is absent from fruits [78,79,80,81]. Yeast has been proved to have an essential nutritional role in *D. suzukii* larval development, survivorship, eclosion rate, and adult body mass [82,83,84]. The mass-rearing protocol of *D. suzukii* is currently based on a wheat bran larval diet that includes 7% inactive brewer’s yeast [44]. Information collected from SIT facilities, currently rearing *C. capitata,* indicate that yeast-related expenses can be very high and, in some cases, they can be as high as 12% of the whole production cost [28]. Therefore, yeast replacement with a cheaper protein source could significantly decrease the cost of a mass-rearing facility. However, this replacement should not compromise the production of high biological quality insects. The yeast substitute should be of equal nutritional value, if not higher. 

Following the promising results of a similar study in *C. capitata*, we tested whether *Enterobacter* sp. AA26 dry biomass could be a reliable alternative protein source. The total replacement of brewer’s yeast had detrimental effects on female fecundity, pupal weight, and adult survivorship and recovery, thus indicating that *Enterobacter* sp. AA26 cannot fulfill the protein requirements of *D. suzukii* when yeast is absent from the diet. Interestingly, the partial yeast replacement did not present severe effects (apart from the adult recovery rate), suggesting that halving the yeast quantity is still sufficient to produce fit adults. In contrast, Kyritsis et al. suggested that *Enterobacter* sp. AA26 dry biomass can be used as an adequate replacement of brewer’s yeast without any negative impact on the biological quality and the productivity rate of *C. capitata* [28]. The different physiology and biology of the two insect species might be one of the reasons why *Enterobacter* sp. AA26 failed to “act” as a suitable protein source. In addition, *Enterobacter* sp. AA26 is a *C. capitata* gut isolate, and although *Enterobacteriaceae* prevail in *D. suzukii* gut and *Enterobacter* sp. has been detected in a few *D. suzukii* studies, no data is available whether this specific AA26 strain is a member of the *D. suzukii* gut microbiota. Strain inconsistency or even bacterial competition could explain why *Enterobacter* sp. AA26 was an unfavorable protein source for *D. suzukii*. A recent study has shown that an infection of *E. ludwigii* in *D. melanogaster* affected the development of the flies and caused age-dependent neurodegeneration, thus indicating that specific *Enterobacter* sp. can negatively impact core aspects of the biology and behavior of the flies [85]. Further studies are required to identify promising microbe candidates with high nutritional value for *D. suzukii*.

Previous studies on fruit flies have clearly indicated that insect fitness is correlated to both the type and dose of the amino acids provided by the diet [86,87]. A study by Azis et al. 2019, compared the amino acid and vitamin content of *Enterobacter* sp. AA26 and Torula yeast [59]. *Enterobacter* sp. AA26 proved to be a sufficient source of all the essential nutrients required by *C. capitata*. Both essential and non-essential amino acids and vitamins were included in adequate amount in *Enterobacter* sp. AA26 biomass thus making it a strong candidate for the replacement of Torula yeast. Although glutamic acid and proline represented a lower fraction of the protein content compared to *Enterobacter* sp. AA26, the overall performance of the diet was equal to the Torula one for *C. capitata* larvae [59]. However, one cannot rule out the possibility that this lower percentage of glutamic acid and proline could affect the *D. suzukii* development and fitness. At the present study, Torula yeast has not been used and therefore it is not safe to extrapolate any conclusions related to the inactive brewer’s yeast used for *D. suzukii*, since the two yeast types have different nutritional properties [88]. 

The outcomes of our study suggest that the “lack of performance” of *Enterobacter* sp. AA26 might not be due to the protein content per se but due to the protein quality. The nutritive value of the yeast’s protein content is quite high and studies on the proteinogenic composition of yeast extracts have shown that the protein proportion of brewer’s yeast can be more than 60% and the proportion of free amino acids more than 30%, thus indicating a rich protein source [88,89]. Apart from the essential amino acids, the content of minerals, vitamins and lipids play a role in the nutrient composition of brewer’s yeast. Significant differences between brewer’s yeast and *Enterobacter* sp. AA26 in the bioavailability of nutrients, as well as in the interactions among individual nutrients could be a reason explaining the reduced performance of *Enterobacter* sp. AA26. To the best of our knowledge there is not a corresponding study that compares *Enterobacter* sp. AA26 with inactive brewer’s yeast, and thus our insight regarding their differences in terms of nutritional value is limited. A future study could shed light on this aspect and reveal why *Enterobacter* sp. AA26 is not sufficient as a protein source for the development of *D. suzukii*. 

## 5. Conclusions

In the context of an SIT application, improvement of the mass-rearing protocols is always in the center of research efforts. Our findings clearly highlighted the importance of yeast as a diet component for *D. suzukii*. The *C. capitata* gut isolate, *Enterobacter* sp. AA26, cannot replace nutritionally the inactive brewer’s yeast in the *D. suzukii* larval diet. However, one should not exclude the possibility that bacterial isolates coming from the *D. suzukii* gut could have a beneficial effect on the fly productivity or be an adequate protein source that would eventually lead in yeast replacement. Future studies should focus on dissecting the gut bacterial diversity of *D. suzukii* and employ the most abundant inhabitants as candidates for the yeast replacement. In addition, cost estimation studies should also be performed for the promising candidates to elucidate their potential as core larval diet components in mass-rearing facilities.

## Data Availability

The data presented in this study are available on request from the authors.
